# Prion Protein Gene Variability in Spanish Goats. Inference through Susceptibility to Classical Scrapie Strains and Pathogenic Distribution of Peripheral PrP^sc^

**DOI:** 10.1371/journal.pone.0061118

**Published:** 2013-04-08

**Authors:** Cristina Acín, Inmaculada Martín-Burriel, Eva Monleón, Jaber Lyahyai, José Luis Pitarch, Carmen Serrano, Marta Monzón, Pilar Zaragoza, Juan José Badiola

**Affiliations:** 1 Centro de Investigación en Encefalopatías y Enfermedades Transmisibles Emergentes, Facultad de Veterinaria, Universidad de Zaragoza, Zaragoza, Spain; 2 Laboratorio de Genética Bioquímica (LAGENBIO), Facultad de Veterinaria, Universidad de Zaragoza, Zaragoza, Spain; 3 Departamento de Anatomía e Histología Humanas, Facultad de Medicina, Universidad de Zaragoza, Zaragoza, Spain; 4 Centre de Génomique Humaine, Faculté de Médecine et de Pharmacie, Université Mohammed V Souissi, Rabat, Morocco; USGS National Wildlife Health Center, United States of America

## Abstract

Classical scrapie is a neurological disorder of the central nervous system (CNS) characterized by the accumulation of an abnormal, partially protease resistant prion protein (PrP^sc^) in the CNS and in some peripheral tissues in domestic small ruminants. Whereas the pathological changes and genetic susceptibility of ovine scrapie are well known, caprine scrapie has been less well studied. We report here a pathological study of 13 scrapie-affected goats diagnosed in Spain during the last 9 years. We used immunohistochemical and biochemical techniques to discriminate between classical and atypical scrapie and bovine spongiform encephalopathy (BSE). All the animals displayed PrP^sc^ distribution patterns and western blot characteristics compatible with classical scrapie. In addition, we determined the complete open reading frame sequence of the *PRNP* in these scrapie-affected animals. The polymorphisms observed were compared with those of the herd mates (n = 665) and with the frequencies of healthy herds (n = 581) of native Spanish goats (Retinta, Pirenaica and Moncaina) and other worldwide breeds reared in Spain (Saanen, Alpine and crossbreed). In total, sixteen polymorphic sites were identified, including the known amino acid substitutions at codons G37V, G127S, M137I, I142M, H143R, R151H, R154H, R211Q, Q222K, G232W, and P240S, and new polymorphisms at codons G74D, M112T, R139S, L141F and Q215R. In addition, the known 42, 138 and 179 silent mutations were detected, and one new one is reported at codon 122. The genetic differences observed in the population studied have been attributed to breed and most of the novel polymorphic codons show frequencies lower than 5%. This work provides the first basis of polymorphic distribution of *PRNP* in native and worldwide goat breeds reared in Spain.

## Introduction

Scrapie is a transmissible spongiform encephalopathy (TSE) that affects domestic small ruminants all around the world. The natural occurrence of the disease in goats is lower than that in sheep; however, the implementation of active surveillance in 2002 demonstrated that the prevalence of this disease was underestimated in this species [1]. In addition, certain novel prion strains, such as Nor98 [2] and BSE [3], have been naturally detected in goats. The first case of scrapie in goats in Spain was diagnosed by the National Reference Centre of TSEs of Zaragoza in 2002 and was found in a pure dairy herd of Alpine and Saanen breeds. Since then, several cases of scrapie in goats have been diagnosed in Spain. Specifically, between 2002 and 2010, fifty-one scrapie outbreaks have been diagnosed, of which 10% are typified as atypical scrapie [4]. The scrapie outbreaks detected involved equally pure goat herds and mixed sheep-goat flocks.

Scrapie in goats, as in sheep, is characterised by deposition of an abnormal, partially protease resistant prion protein (PrP^sc^) in the central nervous system (CNS) and in some peripheral tissues. The capacity to distinguish between classical scrapie and bovine spongiform encephalopathy (BSE) in small ruminants has been important for risk assessments in both agriculture and human health within the European Union and was motivated because of the first description of BSE in a goat by the national French active surveillance [3]. This detection implied changes in political regulations in order to prioritise biochemical differentiation between the two strains [5]. Several biochemical tests have been approved for differentiation between BSE and scrapie [6], and immunohistochemical procedures have been established with the same purposes in lymphoid [7] tissue and the central nervous system [8]. Although peripheral distribution of PrP^sc^ has been largely demonstrated in sheep [9], a specific study of PrP^sc^ distribution in goat peripheral tissues has not yet been performed.

Resistance or susceptibility to the scrapie agent in goats has been studied mostly by European countries where the caprine population is large (France, Italy, United Kingdom or Greece; [10]) or the incidence of scrapie disease in goats is high (such as Cyprus; [11]). These studies have shown that allelic variation of the *PRNP* gene can modulate susceptibility to the scrapie disease [12]. In particular, thirty-seven amino acid substitutions have been described in worldwide goat breeds (W18R, V21A, G22C, L23P, G37V, S39R, G49S, P63L, Q101R, W102G, T110P, G127S, L133Q, M137I, R139S, I142M, I142T, H143R, G145D, N146D, N146S, R151H, R154H, Q163Stop, P168Q, I185F, T194P, F201L, I208T, R211Q, R211G, I218L, T219I, Q220H, Q222K, G232W, P240S [13], [14], [15], [16]), of which, only G127S [15], I142M [12], N146S/D [17], H154R [18], [19], [17], [20], Q211R [20] and Q222K [21], [19], [20] have been related with susceptibility or resistance to scrapie in goats. At least 16 silent mutations have also been found in caprine *PRNP* [13].

Despite the high number of polymorphisms observed in the goat *PRNP* gene, knowledge of their association with scrapie susceptibility or resistance is very limited because the incidence of natural scrapie in goats is underestimated. At present, little is known about the *PRNP* haplotype distribution in Spanish goats. Only a preliminary study presented by our group described some polymorphisms observed in the Spanish goat population [16], [1]. Moreover, whereas *PRNP* polymorphisms in scrapie-infected sheep bred in Spain are well known [22], [23], [24], the variation in the coding region of caprine *PRNP* in Spanish goats with scrapie has never been investigated. This work has been focused on worldwide goat breeds (Saanen and Alpine) and three Spanish native breeds (Moncaina, Pirenaica and Retinta) that could have new variants that contribute to the knowledge of the caprine scrapie genetic background.

The Swiss Alps seem to be the origin of the Alpine and Saanen breeds, which spread all along the French Alps and crossed with several native breeds. These two breeds are probably the most cosmopolitan worldwide breeds, extending along five different continents. In Spain, the first reported Alpine herds came from France and settled in Castilla y Leon, afterwards spreading into the northern part of Spain (Navarra, Asturias and Catalonia). The origin of the Pirenaica breed is placed in Central Europe. Ancestral animals settled in the Pyrenees and spread out to other mountain areas in the Iberian Peninsula. The Moncaina breed is an endangered goat descended from the Pirenaica goat. A possible Alpine origin has been proposed for the Retinta goat. The concept of breed applied to domestic goats could be meaningful at the phenotypical or productive level, but it seems to be ambiguous and diffuse at the mitochondrial DNA level [25] as mitochondrial DNA studies have revealed the weak phylogeographical structure of most domestic goat breeds [26], including Iberian Spanish goat breeds [27].

The aims of this study were: a) To describe the peripheral distribution of PrP^sc^ in Spanish scrapie-affected goats; b) To differentiate, through immunohistochemical and biochemical methodologies, the strains causing the different scrapie cases (classical and atypical scrapie and BSE); c) To determine the *PRNP* genotypes of scrapie affected animals and healthy herd mates from four scrapie-affected flocks in a case-control study; and d) To compare these results with the *PRNP* variants observed in healthy animals from breed survey.

## Materials and Methods

### Animals and samples

#### Ethics statement

This study was performed in accordance with the recommendations for the care and use of experimental animals of the University of Zaragoza (R.D. 1201/2005). The Committee on the Ethics of Animal Experiments approved the protocol (Permit Number: PI12/11).

#### Natural scrapie-affected herds (Case-control study)

In the framework of the active and passive surveillances developed in Spain, thirteen goat scrapie cases were diagnosed between 2002 and 2009. Six of these cases were detected because of active surveillance (primary case or eradication measures) and seven because of passive surveillance (clinical signs). These scrapie-infected goats were detected in Saanen, Alpine and crossbreed herds, eleven of which belonged to mixed flocks of sheep and goats and two to pure goat herds. The age of the animals varied between 5–6 years (11 out of 13) and the two goats left were older than 8 years old.

The Spanish national scrapie surveillance program provides no possibility of applying genotyping measures in goats, as the entire scrapie-affected herd is sacrificed. Nevertheless, our research group applied lymphoid tissue biopsies to detect live scrapie-affected goats. Goats with scrapie positive biopsy and/or clinically affected were removed from the flock and monitored until the appearance of clinical signs (three years on average in the case of positive biopsies) in our research facilities. When the clinical signs progressed to severe levels, the animals were euthanized by intravenous injection of sodium pentobarbital and exsanguination.

*Biopsies:* Goats involved in four outbreaks of scrapie from Asturias and Aragón were biopsied from the third eyelid and/or RAMALT (rectoanal mucosa-associated lymphoid tissue). Both tissues were analyzed indistinctly and their results were consistent. Specifically, 180 biopsies were obtained from pure herds and 30 from mixed flocks. For the third eyelid biopsies, 5 mm of lymphoid tissue from the bilateral patches of the third eyelid was sliced and subsequently formalin fixed [28]. For the RAMALT biopsies, 1 cm of lymphoid tissue from the rectoanal mucosa was sliced and formalin fixed for preclinical scrapie diagnosis [29].

*Scrapie diagnosis:* The medulla oblongata (11) and tonsils (1) were sampled and analyzed by immunohistochemistry (IHC) for post-mortem diagnosis of scrapie. At the time of writing this paper, one scrapie-affected goat was still alive, and scrapie diagnosis in this case was performed by RAMALT biopsy.

*Samples from peripheral tissues:* Six scrapie cases in which the whole body was available were selected to study the peripheral PrP^sc^ distribution. At necropsy, the samples recorded were: nervous tissue (cervical, thoracic and lumbar spinal cord; obex; pons; midbrain; cerebellum; frontal, parietal and occipital cortex; striatum; thalamus; hippocampus; trigeminal ganglia; pituitary gland; optic chiasm; eye; olfactory bulb and nasal mucosa); lymphoreticular tissue (3^rd^ eyelid; tonsils; lymph nodes [retropharyngeal, mediastinal, mesenteric, iliac, axillary, pre-scapular, submandibular, popliteal and mammary]; ileal and jejunal Peyer patches; ileocaecal valve and spleen); digestive tissue (tongue, oesophagus, rumen, reticulum, omasum, abomasum, duodenum, jejunum, ileum, caecum, rectum, liver and pancreas); other tissues (lung, heart, skin, kidney, urinary bladder, adrenal gland, striated muscle, uterus, ovaries and mammary gland).

*Blood:* Additionally, a representative number of blood samples were obtained from each scrapie-affected herd (random sampling system, higher than 50% of the flock in all cases) before slaughter: 51 goats from the Alpine scrapie-affected herd (SA), 52 from the Saanen scrapie-affected herd (SS), 10 from the crossbreed scrapie-affected herd 1 (SC1) and 552 from the crossbreed scrapie-affected herd 2 (SC2).

#### Native healthy herds

Blood samples from native breeds from the Aragón region (Moncaina and Pirenaica) and from the Extremadura region (Retinta) and two worldwide breeds (Saanen and Alpine) from Asturias, the same region as the scrapie-affected herd, were selected for genotyping studies. Five hundred and eighty-one samples were obtained from eight healthy herds. The animals analyzed were 39 Alpine (1 herd, A1); 42 Saanen (1 herd, S1); 386 Moncaina (2 herds, M1 and M2); 43 Pirenaica (2 herds, P1 and P2); and 71 Retinta (2 herds, R1 and R2).

### PrP^sc^ immunochemical determination

#### PrP^sc^ immunohistochemical detection

Four-micrometre sections of formaldehyde-fixed and paraffin-wax-embedded lymphoid tissues (in the case of the biopsies) and medulla oblongata tissues (in the case of post-mortem surveillance) were subjected to immunohistochemical diagnosis for scrapie using the monoclonal antibody L42 (1/500; R-Biopharm). The technical description has been described elsewhere [30]. Apart from the CNS, the distribution of PrP^sc^ was evaluated in ten different tissues (see above) by immunohistochemistry, using the monoclonal antibody L42 as described previously [30].

#### Mapping of antigenic sites of caprine PrP^sc^ by immunohistochemistry

With the aim to differentiate among classical and atypical scrapie and BSE, different specific PrP^sc^ monoclonal antibodies were applied in the obex from eleven scrapie-infected goats. In five of these goats, the mapping epitope was also developed in the lymphoid (tonsil) tissue. These antibodies show different intra-neuronal or intra-macrophage truncation patterns for scrapie and BSE, as described by other authors in sheep [31] and goats [32], as well as differences in the morphology of the PrP^sc^ granules in the tingible body macrophages (TBM) when antibodies directed towards amino acids 93–106 are used [7], [31].

The monoclonal antibodies were kindly supplied by the Central Veterinary Institute of Wageningen UR, Department of Bacteriology and TSE from Lelystad: R522-7 [1/2000; epitope site 94–105], with high affinity to scrapie PrP^sc^ and less affinity to BSE PrP^sc^ (no intraneuronal PrP^sc^ deposition staining in BSE); 12B2 [0.2 µg/ml; 97–115], with high affinity to scrapie PrP^sc^ and less affinity to BSE PrP^sc^ (no intraneuronal PrP^sc^ deposition in BSE); 1E4 [2.5 µg/ml; 108–119], with high affinity to BSE and scrapie PrP^sc^ (intraneuronal PrP^sc^ in both diseases); and 6C2 [1∶50; 117–130] with high affinity to BSE and scrapie PrP^sc^ (intraneuronal PrP^sc^ in both diseases). In addition, P4 (1/500; R-Biopharm; 93–99) was also included in the epitope study because of the absence of intraneuronal PrP^sc^ detection in BSE-infected tissues [8]. The immunohistochemical procedure has been described elsewhere [30]. Confirmed positive sheep scrapie and bovine BSE samples were also included as positive controls to ensure the PrP^sc^ antibody affinity and the sensitivity of the technique. As negative controls, brain tissues from scrapie-unexposed sheep and scrapie-affected brain tissues without the primary antibody were also processed.

In the nervous tissue, the obex was the brain area analysed, differentiating seven anatomical sites: the dorsal motor nucleus of the vagus (DMNV); hypoglossal and lateral cunneate nuclei; spinal tract of the trigeminal nerve; reticular formation; raphe and olivary nuclei. The intraneuronal PrP^sc^ was scored from 0 (absence) to 3 (maximum presence) in steps of 0.5. Only the scores between 0 and 1 varied between 0.2 (minimum presence) and 0.3 (more than 1 neuron stained but less than 5).

In the lymphoid tissue, the tonsil was antibody mapped to label PrP^sc^ in the tingible body macrophages to differentiate between scrapie and BSE.

#### Differentiation of prion strains by immunoblotting

Eleven out of 13 goats were subjected to the VLA hybrid western blot method to differentiate between scrapie and BSE. In addition, the CEA discriminatory test for strain typing of transmissible spongiform encephalopathies in small ruminants was developed to differentiate between classical and atypical scrapie [6].

### *PRNP* sequencing analysis

#### DNA extraction

*Blood:* Genomic DNA was extracted using the GFX™ Genomic Blood DNA purification kit (GE Healthcare Bio-Sciences AB, Uppsala, Sweden), following the manufacturer's recommendations.

*Brain:* DNA was extracted from 500 mg of frozen brain. After mechanical disruption using liquid nitrogen and a pestle, the tissue was immersed in a solution of 180 µl ATL buffer (Qiagen) and 20 µl proteinase K (200 mg ml^−1^; Qiagen). After undergoing vigorous vortexing for 1 min, the sections were incubated overnight at 56°C. After overnight digestion, the DNA was purified using a standard phenol/chloroform/isoamyl alcohol (25∶ 24∶ 1) treatment. The ethanol-precipitated DNA pellet was dissolved in 50 µl TE buffer (Qiagen).

#### *PRNP* genotype analysis

The complete *PRNP* open reading frame was sequenced in 1259 goats, including 13 scrapie-affected goats, 665 goats from scrapie-affected herds (AS, SS, CS1 and CS2) and 581 goats from native healthy herds (A1, S1, M1, M2, P1, P2, R1 and R2).

The complete coding region was amplified using the following primers: p8 (fwd) (5′-CAGGTTAACGATGGTGAAAAGCCACATAGG-3′) and p9 (rev) (5′-GGAATTCTATCCTACTATGAGAAAAATGAGG- 3′) [33].

The PCR fragments were purified using the MALDIspot™ Kit and a vacuum manifold from Millipore® and sequenced with the Big Dye Kit® from Applied Biosystems. The same PCR primers were used for bi-directional sequencing, and the chromatograms were analysed using BioEdit v. 4.8.6 [34].

To discriminate the haplotypes presented by double-heterozygote individuals for codons 240 and 154, the alleles P240 and S240 were independently amplified using allele-specific PCR (AS-PCR) and further sequenced as described previously [16].

#### Statistical analysis

Genotypic distributions for the different populations were compared statistically using the χ^2^ test for independence using the Yates correction for continuity for N×K contingency tables. *P*<0.05 was considered statistically significant. The haplotypic and genotypic frequencies for each population were calculated using the GENEPOP program [35]. This program was also used to perform a statistical test to determine possible deviations from the Hardy-Weinberg proportion. A Markov chain method was applied to calculate the exact P-values; the length of the chain was set to 10^5^ iterations. Chi-squared statistic-treating alleles rather than genotypes as individual entities are appropriate only when the Hardy-Weinberg equilibrium holds [36]. To avoid a possible flock effect, before pooling together the data for breed comparison, the distributions of genotypic frequencies were compared between each flock belonging to the same breed using the χ^2^ test for independence and the Yates correction for continuity for N×K contingency tables.

## Results

### Diagnosis, peripheral distribution and differentiation of prion strains

#### Clinical signs

In 7 out of 13 goats, clinical signs associated with scrapie could be evaluated. From these seven animals, 5 where previously detected by third eyelid and/or RAMALT biopsy and were allowed to progress to clinical disease. From highest to lowest, the disease was characterised by ataxia, incoordination and thinness>lethargy and trembling>pruritus and alopecia>lameness>recumbency and hyperexcitability.

#### Immunohistochemistry of third eyelid and/or RAMALT biopsy

Biopsies were assessed in 7 out of the 13 scrapie-affected goats. In 5 cases, PrP^sc^ could be detected in the lymphoid tissue associated to rectal mucosa or in the third eyelid.

#### Scrapie post-mortem diagnosis

PrP^sc^ was detected by immunohistochemistry in the obex region of 11 scrapie-affected goats and in the lymphoid tissue of one scrapie-affected goat. In the CNS, the PrP^sc^ deposition was mainly confined to the dorsal motor nucleus of the vagus and the spinal tract of the trigeminal nerve.

#### Detection of PrP^sc^ in peripheral organs

PrP^sc^ was detected in the tonsils, iliac, axillary, pre-scapular, submandibular, popliteal, and mammary lymph nodes of the six goats analyzed, but just in five in the mediastinal and mesenteric lymph nodes, ileal and jejunal Peyer patches, ileocaecal valve and spleen. The enteric nervous system was positive throughout the whole intestine, from duodenum to rectum, in the five animals in which PrP^sc^ was also detected in all the areas of the CNS (see Table 1).

**Table 1:** Distribution of PrP^sc^ through nervous, lymphoreticular and other tissues by immunohistochemistry (Mab L42; 1/500; R-Biopharm).

Nervous tissue	C1	C2	C3	C4	C5	C6
CNS *	−	+	+	+	+	+
Hippocampus	−	+	+	+	+	−
Trigeminal ganglia	−	nd	−	−	+	+
Pituitary gland	−	+	−	−	+	+
Optic chiasm	−	+	+	+	+	+
Eye	−	−	+	−	+	−
Olfactory bulb	−	+	+	+	+	+
Olfactory mucosa	−	+	+	−	nd	nd
**LRS**						
3^rd^ eyelid	−	+	nd	nd	+	nd
Tonsils	+	+	+	+	+	+
Retropharyngeal L	+	+	+	+	+	+
Mediastinal L	+	+	+	+	−	+
Mesenteric L	−	+	+	+	+	+
Other lymph nodes**	+	+	+	+	+	+
Ileal PP	−	+	+	+	+	+
Jejunal PP	nd	+	+	+	+	+
Ileocaecal valve	−	+	+	+	+	+
Spleen	−	+	+	+	+	+
**Other tissues**						
Oesophagus	−	−	−	−	−	−
Ru, Re, Om, Abo	−	−	−	−	−	−
Duo, Je, Ile, Cae, Co, Rec (ENS)	−	+	+	+	+	+
Liver	−	−	−	−	−	−
Pancreas	−	−	−	−	−	−
Lung	−	−	−	−	−	−
Muscle	−	−	−	−	−	−
Tongue	−	nd	+	−	−	−
Uterus	−	−	−	−	−	−
Ovaries	−	−	−	−	−	−
Mammary gland	−	−	−	−	−	−
Kidney	nd	−	−	−	−	−
Urinary bladder	−	−	−	−	−	−
Adrenal gland	+	−	+	nd	+	+
Heart	−	−	−	−	−	−
Skin	−	−	−	−	−	−
**Age (years)**	6	6	5	5	5	5
**Haplotype variant**	2/15	1/2	1/1	1/1	1/5	1/1

The age of the animals as well as the combination of haplotype variants is also indicated.

CNS: Central nervous system; L: Lymph node; LRS: Lymphoreticular system; PP: Peyer patches; Ru: Rumen; Re: Reticulum; Om: Omasum; Abo: Abomasum; Duo: Duodenum; Je: Jejunum; Ile: Ileum; Cae: Caecum; Co: Colon; Rec: Rectum; ENS: Enteric nervous system.

* CNS: Cervical, Thoracic, Lumbar spinal cord; Obex; Pons; Midbrain; Cerebellum; Frontal, Thalamic and Occipital cortex; Striatum; Thalamus.

** Other Lymph nodes: Iliac; Axillary; Pre-scapular; Submandibular; Popliteal; Mammary.

Nd: Not done.

**Figure 1:**
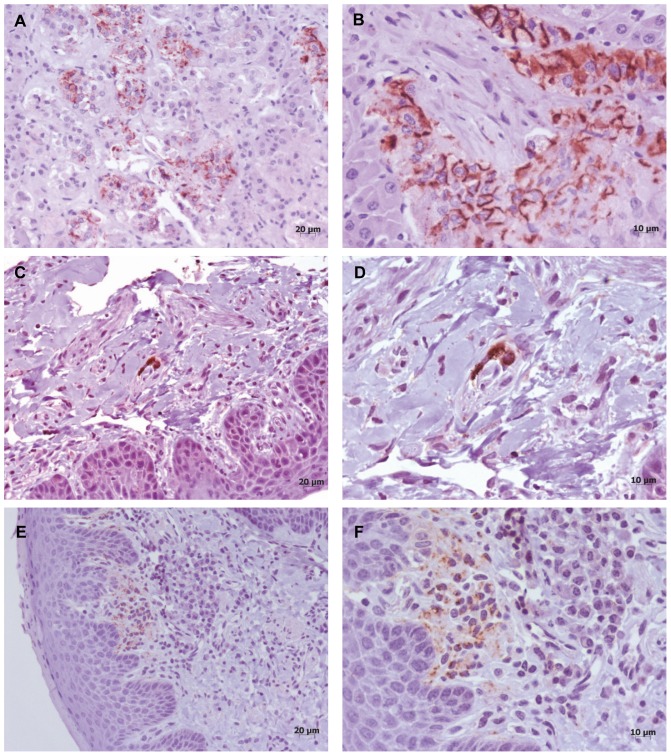
Immunohistochemical distribution of PrP^sc^ through peripheral tissues. mAb L42. A) PrP^sc^deposits detected in the medullar region of the adrenal gland. B) Detail of PrP^sc^ distribution in the fascicular region of the adrenal gland. C) and D) Caudal vestibular region of the nasal mucosa. PrP^sc^ deposits in a spin bundle of the submucosa. E) and F) Tongue. PrP^sc^ deposits in the *lamina propria* of the mucosa.

Immunohistochemical detection of PrP^sc^ was also observed in other organs that have previously been described as positive for ovine scrapie, such as the adrenal gland [37], the olfactory mucosa [38] and the tongue [39]. In the adrenal gland, PrP^sc^ was detected in the fascicular region of the cortex as well as in the medullar chromaffin cells. The nasal mucosa showed mild deposition of the PrP^sc^, which was limited to some isolated axon bundles; in contrast, in the tongue, PrP^sc^ was detected in aggregated clusters of lymphocytes (see Figure 1).

#### Mapping of antigenic sites on the caprine PrP by PrP-specific antibodies

**Figure 2:**
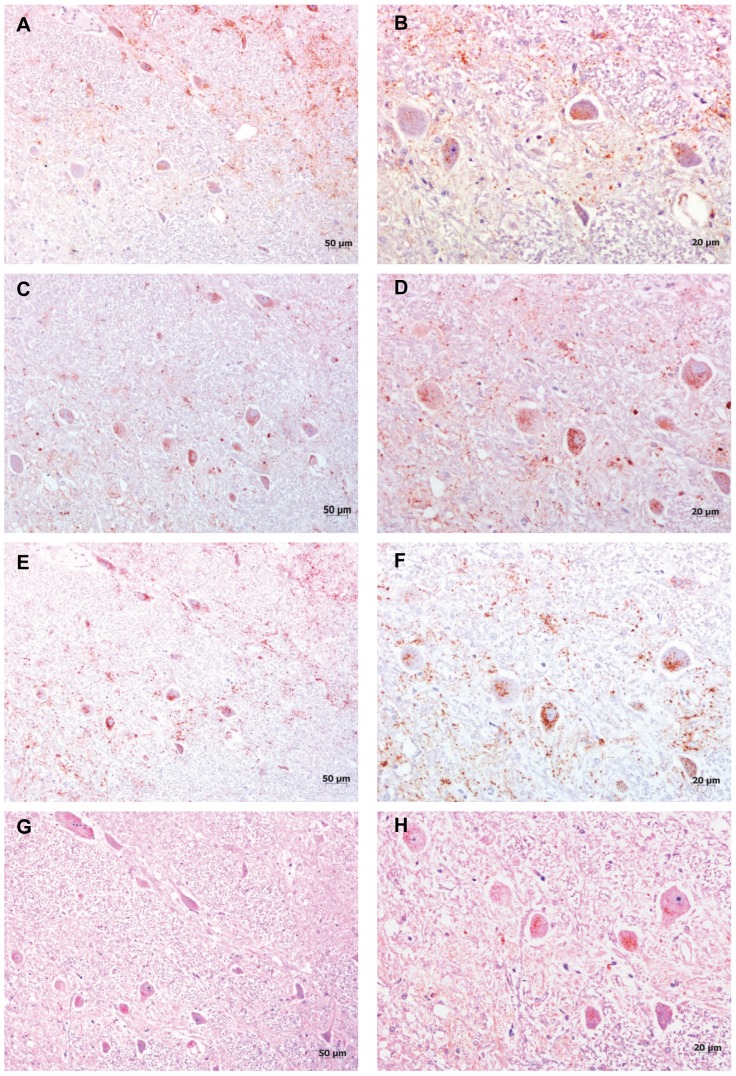
Intraneuronal deposition of PrP^sc^ by immunohistochemistry. Maintenance of intraneuronal deposits with the different epitope mapping antibodies. Lateral cunneate nucleus. A) and B) mAb P4; C) and D) mAb R-522; E) and F) mAb 12B2 G) and H) mAb 6C2.

**Figure 3:**
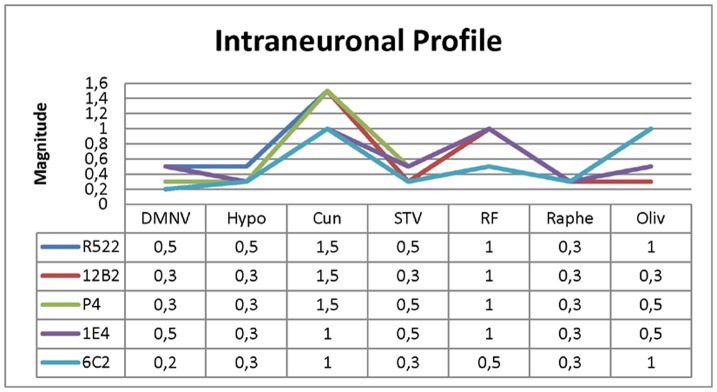
Intraneuronal PrP^sc^ immunohistochemical profile in the obex, a representative profile is shown (C2). Maintenance of PrP^sc^ intraneuronal profile through all analysed antibodies. DMNV: Dorsal Motor Nucleus of the Vagus; Hypo: Hypoglossal nucleus; Cun: Lateral cunneate nucleus; STV: Spinal tract of the trigeminal nerve; RF: Reticular formation; Raphe and Oliv: Olivary nuclei.

*Obex:* The intraneuronal PrP^sc^ profile showed the same phenotype for the five antibodies (see Figure 2) in all the animals studied. Nevertheless, some differences have been detected that could be associated with high or low magnitudes of PrP^sc^ deposition because of the status of the clinical disease. Figure 3 shows that the antibodies R-522, 12B2, P4, IE4 and 6C2 provide a similar profile, nevertheless, 6C2 antibody showed less PrP^sc^ immunoaffinity and slightly more background than the other antibodies, what could have interfered in the definition of the specific phenotype.

*Tonsil lymphoid tissue:* All the animals showed clusters of PrP^sc^ granules in the macrophages of the tingible body. Tingible body macrophage labelling was maintained with each antibody used, which is incompatible with BSE infection. A diagnosis of atypical scrapie is similarly inconsistent with lymphoid tissue accumulation of PrP^sc^ (data not shown).

#### Differentiation of prion strains by immunoblotting

**Figure 4:**
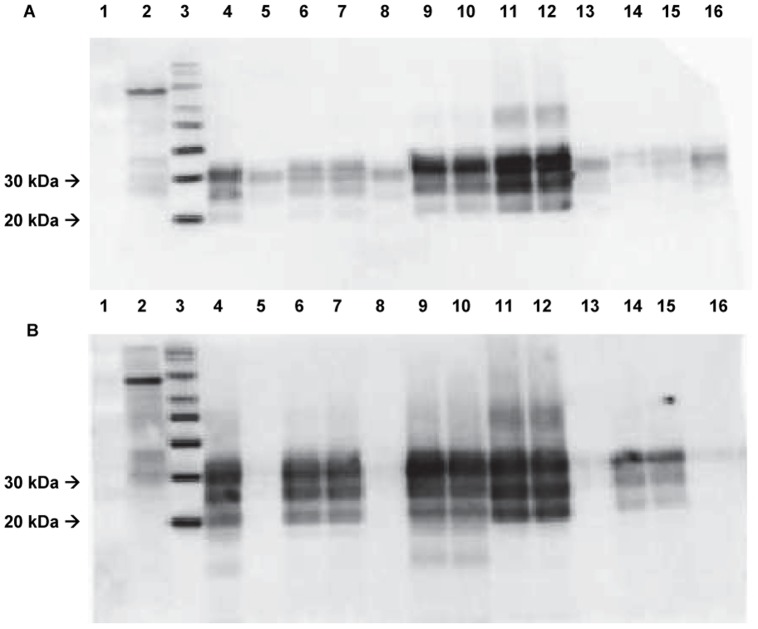
VLA Discriminatory western blot (WB) showing samples from C2 to C5 (brain stem) in duplicate lanes. A) The antibody used to detect PrP^sc^ was mAb 6H4. Lane 1: Empty; Lanes 2 and 3: Biotinylated molecular weight marker and MagicMark™ XP western protein standard respectively; Lane 4: Classical scrapie +ve; Lanes 5, 8, 13 and 16: BSE +ve; Lanes 6 and 7: C2 classical scrapie +ve; Lanes 9 and 10: C3 classical scrapie +ve; Lanes 11 and 12: C4 classical scrapie +ve; Lanes 14 and 15: C5 classical scrapie +ve. B) The antibody used to detect PrP^sc^ was mAb P4. Lane 1: Empty; Lanes 2 and 3: Biotinylated molecular weight marker and MagicMark™ XP western protein standard respectively; Lane 4: Classical scrapie +ve; Lanes 5, 8, 13 and 16: BSE +ve; Lanes 6 and 7: C2 classical scrapie +ve; Lanes 9 and 10: C3 classical scrapie +ve; Lanes 11 and 12: C4 classical scrapie +ve; Lanes 14 and 15: C5 classical scrapie +ve.

**Figure 5:**
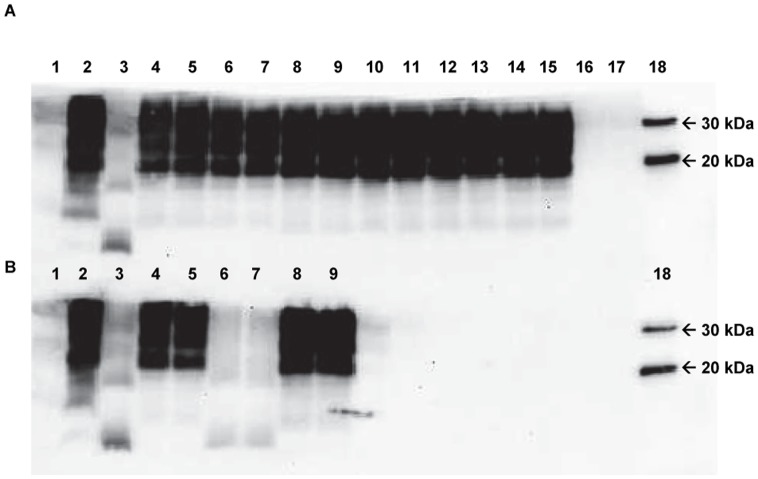
Bio-Rad western blot (WB) showing samples from C2 to C9 (brain stem) in duplicate lanes. The antibody used to detect PrP^sc^ was mAb Sha31 as provided by the protocol test. Relative molecular mass (Mr) for MagicMark markers are shown on the right. A) Lane 1: Kaleidoscope; Lane 2: Classical scrapie +ve; Lane 3: Atypical scrapie +ve; Lanes 4 to 15: C2 to C7 classical scrapie +ve in duplicate lanes; Lanes 16 and 17: Scrapie −ve; Lane 18: MagicMark XP western standard. B) Lane 1: Kaleidoscope; Lane 2: Classical scrapie +ve; Lanes 3, 6 and 7: Atypical scrapie +ve; Lanes 4 and 5: C8 classical scrapie +ve; Lanes 8 and 9: C9 classical scrapie +ve; Lanes 10 to 17: Empty; Lane 18: MagicMark XP western standard.

Classical scrapie is characterised by the presence of diglycosylated, monoglycosylated and nonglycosylated immunochemical bands, detected by western blot [6]. In addition, both the P4 and 6H4 antibodies have the ability to detect this classical profile, which is not the case if BSE is present. Our results showed that the PrP glycotype profile was not compatible with BSE in any of the 11 scrapie-affected goats analysed by the VLA-Hybrid western blot. The BioRad-discriminating western blot did not show the presence of atypical scrapie in the analysed goats either (see Figures 4 and 5).

### *PRNP* variation in goats

The coding region of the caprine *PRNP* gene was sequenced in 1259 goats. Genetic variation leading to amino acid substitutions was observed in 16 codons (see Table 2). The amino acids are described in one letter codes (e.g., Serine: S). The codon position is given after the letter; polymorphisms are shown as, e.g., R211Q.

**Table 2:** Allelic frequencies of *PRNP* polymorphisms (given as a fraction of unity) in native herds, healthy goats in scrapie-affected herds and scrapie-affected goats.

Allele		aa	Breeds												
			M.1	M.2	P.1	P.2	R.1	R.2	A.1	S.1	SA	SS	SC1	SC2	Pos
Total			40	346	21	22	16	55	39	42	51	52	10	552	13
**37**	**GGG**	**G**	0.975	0.986	1	1	1	1	1	1	1	1	1	0.996	1
	**GTG**	**V**	0.025	0.014	0	0	0	0	0	0	0	0	0	0.004	0
**74**	**GGT**	**G**	1	1	1	1	1	0.964	1	1	1	1	1	1	1
	**GAT**	**D**	0	0	0	0	0	0.036	0	0	0	0	0	0	0
**112**	**ATG**	**M**	1	1	1	1	1	1	1	1	1	1	1	1	0.962
	**ACG**	**T**	0	0	0	0	0	0	0	0	0	0	0	0	0.038
**127**	**GGC**	**G**	0.988	1	1	1	1	1	1	0.976	0.951	1	1	1	0.962
	**AGC**	**S**	0.012	0	0	0	0	0	0	0.024	0.049	0	0	0	0.038
**137**	**ATG**	**M**	1	0.999	1	1	1	1	1	1	1	1	1	0.995	1
	**ATA**	**I**	0	0.001	0	0	0	0	0	0	0	0	0	0.005	0
**139**	**AGG**	**R**	0.988	0.999	1	1	1	1	1	1	1	1	1	1	1
	**AGC**	**S**	0.012	0.001	0	0	0	0	0	0	0	0	0	0	0
**141**	**CTT**	**L**	1	0.999	1	1	1	1	1	1	1	1	1	1	1
	**TTT**	**F**	0	0.001	0	0	0	0	0	0	0	0	0	0	0
**142**	**ATA**	**I**	0.938	0.874	0.619	0.636	0.875	0.782	0.962	0.798	0.941	0.933	0.850	0.839	0.846
	**ATG**	**M**	0.062	0.126	0.381	0.364	0.125	0.218	0.038	0.202	0.059	0.067	0.150	0.161	0.154
**143**	**CAT**	**H**	1	0.999	1	1	1	1	1	1	1	1	1	1	1
	**CGT**	**R**	0	0.001	0	0	0	0	0	0	0	0	0	0	0
**151**	**CGT**	**R**	1	0.997	1	1	1	1	1	1	1	1	1	1	1
	**CAT**	**H**	0	0.003	0	0	0	0	0	0	0	0	0	0	0
**154**	**CGT**	**R**	0.925	0.860	0.881	0.864	0.969	0.991	0.974	0.988	0.951	1	0.850	0.923	1
	**CAT**	**H**	0.075	0.140	0.119	0.136	0.031	0.009	0.026	0.012	0.049	0	0.150	0.077	0
**211**	**CGA**	**R**	0.875	0.877	0.929	0.932	0.750	0.718	0.897	0.940	0.824	0.750	1	0.821	0.885
	**CAA**	**Q**	0.125	0.123	0.071	0.068	0.250	0.282	0.103	0.060	0.176	0.250	0	0.179	0.115
**215**	**CAA**	**Q**	0.988	0.987	1	1	0.969	1	1	1	1	1	1	1	1
	**CGA**	**R**	0.012	0.012	0	0	0.031	0	0	0	0	0	0	0	0
**222**	**CAG**	**Q**	1	0.997	1	1	1	1	0.936	0.988	0.971	1	1	1	1
	**AAG**	**K**	0	0.003	0	0	0	0	0.064	0.012	0.029	0	0	0	0
**232**	**GGG**	**G**	1	1	1	1	1	1	1	1	0.990	0.990	1	1	1
	**TGG**	**W**	0	0	0	0	0	0	0	0	0.010	0.010	0	0	0
**240**	**CCC**	**P**	0.562	0.627	0.881	0.932	0.719	0.636	0.551	0.655	0.402	0.519	0.550	0.640	0.692
	**TCC**	**S**	0.438	0.373	0.119	0.068	0.281	0.364	0.449	0.345	0.598	0.481	0.450	0.360	0.308

aa. Aminoacid; M. Moncaina; P. Pirenaica; R. Retinta; A. Alpine; S. Saanen; S.A. Scrapie Alpine; S.S. Scrapie Saanen; S.C. Scrapie Crossbreed; Pos. Positive scrapie.

New polymorphisms were found at codons G74D, M112T, R139S, L141F and Q215R. In addition, silent mutations were observed at codons 42, 122, 138 and 179 (data not shown). Considering only the polymorphisms with amino acid changes, 18 haplotypes were observed in the joint populations (see Table 3), of which haplotype 1 was the most common. Haplotypes are presented as groups of amino acids in the order of their codons: 37, 74, 112, 127, 137, 139, 141, 142, 143, 151, 154, 211, 215, 222, 232 and 240. Genotypes are presented as combinations of the haplotype codes as described above.

**Table 3:** Haplotypes detected in the analyzed population.

	Goat *PRNP* codons															
Haplotype	37	74	112	127	137	139	141	142	143	151	154	211	215	222	232	240
**1**	G	G	M	G	M	R	L	I	H	R	R	R	Q	Q	G	P
**2**	G	G	M	G	M	R	L	I	H	R	R	R	Q	Q	G	**S**
**3**	**V**	G	M	G	M	R	L	I	H	R	R	R	Q	Q	G	**S**
**4**	G	**D**	M	G	M	R	L	I	H	R	R	R	Q	Q	G	**S**
**5**	G	G	**T**	G	M	R	L	I	H	R	R	R	Q	Q	G	**S**
**6**	G	G	M	**S**	M	R	L	I	H	R	R	R	Q	Q	G	P
**7**	G	G	M	G	**I**	R	L	I	H	R	R	R	Q	Q	G	P
**8**	G	G	M	G	M	**S**	L	I	H	R	R	R	Q	Q	G	P
**9**	G	G	M	G	M	R	**F**	I	H	R	R	R	Q	Q	G	P
**10**	G	G	M	G	M	R	L	**M**	H	R	R	R	Q	Q	G	P
**11**	G	G	M	G	M	R	L	I	**R**	R	R	R	Q	Q	G	**S**
**12**	G	G	M	G	M	R	L	I	H	**H**	R	R	Q	Q	G	P
**13**	G	G	M	G	M	R	L	I	H	R	**H**	R	Q	Q	G	**S**
**14**	G	G	M	G	M	R	L	I	H	R	**H**	R	Q	Q	G	P
**15**	G	G	M	G	M	R	L	I	H	R	R	**Q**	Q	Q	G	**S**
**16**	G	G	M	G	M	R	L	I	H	R	R	R	**R**	Q	G	P
**17**	G	G	M	G	M	R	L	I	H	R	R	R	Q	**K**	G	**S**
**18**	G	G	M	G	M	R	L	I	H	R	R	R	Q	Q	**W**	**S**
**Human codon correspondence**	**34**	**71**	**109**	**123**	**134**	**136**	**138**	**139**	**140**	**148**	**151**	**208**	**212**	**219**	**229**	**237**

G. Glycine; M. Methionine; R. Arginine; L. Leucine; I. Isoleucine; H. Histidine; Q. Glutamine; P. Proline; S. Serine; V. Valine; D. Aspartic Acid; T. Threonine; F. Phenylalanine; K. Lysine; W. Tryptophan.

The segregation of the polymorphisms detected depended on the selected allelic variant. Whereas V37, D74, F141, R143, Q211, K222 and W232 segregate with serine at 240 position, T112, S127, I137, S139, M142, H151 and R215 segregate with proline at 240 position, and H154 segregates with both P and S240.

#### *PRNP* polymorphisms in the scrapie-affected goats

The polymorphisms detected in the 13 scrapie-affected goats were located at codons M112T, G127S, I142M, R211Q and P240S (see Table 4). Two silent mutations were also observed at codons 42 (cca→ccg) and 138 (agt→agc) in 5 out of 13 scrapie-affected animals. Neither the short octapeptide repeat allele nor the remaining polymorphisms related to scrapie susceptibility, reported by other authors, were observed. In addition to the ancestral haplotype, variants 2, 5, 6, 10 and 15 were observed in the scrapie-affected animals.

**Table 4:** *PRNP* genotypes of the 13 scrapie affected goats.

Scrapie affected Goat	Codon genotype				
	112	127	142	211	240
1	MM	GG	II	RQ	SS
2	MM	GG	II	RR	PS
3	MM	GG	II	RR	PP
4	MM	GG	II	RR	PP
5	MT	GG	II	RR	PP
6	MM	GG	II	RR	PP
7	MM	GG	II	RR	PP
8	MM	GG	II	RQ	PS
9	MM	GG	II	RR	PP
10	MM	GS	II	RR	PS
11	MM	GG	II	RQ	PS
12	MM	GG	MM	RR	PP
13	MM	GG	MM	RR	PP

M. Methionine; G. Glycine; I. Isoleucine; R. Arginine; P. Proline; S. Serine; Q. Glutamine; T. Threonine.

#### Four herd case-control study

The genetic variation observed in the healthy animals of the outbreak herds were located at positions G37V, G127S, M137I, I142M, R154H, R211Q, Q222K, G232W and P240S. The allelic distribution of the polymorphisms detected is shown in Table 2. The polymorphism at codon 37 (G→V) was detected only in the crossbreed population, and the V37 allele frequency was very low (0.004), as well as I137 allele frequency. The described polymorphisms at codons 142 (I→M), 154 (R→H), 211 (R→Q) and 240 (P→S) were the most variable ones in this subpopulation, with observed frequencies ranging between 0.049 and 0.598. The previously reported polymorphisms at codons 127 (G→S) and 222 (Q→K) were observed just in the Alpine affected herd, and the variation at codon 232 (G→R) was almost exclusively found in the Saanen and Alpine affected herds. In addition to the mutations leading to amino acid change, the two silent mutations at codons 42 (ccg→cca) and 138 (agt→agc) were observed in both breeds, and the one at codon 201 (ttc→ttt) was observed in the Alpine goats. Finally, 10 out of the 18 possible haplotypes (see Table 5) were detected in these herds.

**Table 5:** Haplotypic frequencies (%) of *PRNP* polymorphisms in native herds and healthy goats in scrapie-affected herds compared to scrapie-affected goats.

Haplotype	Breeds												
	M.1	M.2	P.1	P.2	R.1	R.2	A.1	S.1	SA	SS	SC1	SC2	Pos
**Total** *	**40**	**346**	**21**	**22**	**16**	**55**	**39**	**42**	**51**	**52**	**10**	**552**	**13**
**1**	41.3	37.6	38.1	45.5	53.1	40.9	48.7	42.9	29.4	45.2	40	41.9	54.2
**2**	26.3	20.1	4.76	0	3.12	4.55	28.2	26.2	33.3	22.1	30	16.1	16.7
**3**	2.5	1.45	0	0	0	0	0	0	0	0	0	0.45	0
**4**	0	0	0	0	0	3.64	0	0	0	0	0	0	0
**5**	0	0	0	0	0	0	0	0	0	0	0	0	4.17
**6**	1.25	0	0	0	0	0	0	2.38	3.92	0	0	0	4.17
**7**	0	0.14	0	0	0	0	0	0	0	0	0	0.45	0
**8**	1.25	0.14	0	0	0	0	0	0	0	0	0	0	0
**9**	0	0.14	0	0	0	0	0	0	0	0	0	0	0
**10**	6.25	12.3	38.1	36.4	12.5	21.8	3.85	20.2	5.88	6.73	15	16	8.33
**11**	0	0.14	0	0	0	0	0	0	0	0	0	0	0
**12**	0	0.29	0	0	0	0	0	0	0	0	0	0	0
**13**	2.5	2.75	0	0	0	0	1.28	1.19	4.9	0	15	1.54	0
**14**	5	11.3	11.9	11.4	3.12	0.91	1.28	0	0	0	0	5.89	0
**15**	12.5	12.1	7.14	6.82	25	28.2	10.3	5.95	17.7	25	0	17.7	12.5
**16**	1.25	1.3	0	0	3.12	0	0	0	0	0	0	0	0
**17**	0	0.29	0	0	0	0	6.41	1.19	2.94	0	0	0	0
**18**	0	0	0	0	0	0	0	0	1.96	0.96	0	0	0
**χ2**	7.19	5.86	11.83	19.88	11.70	11.19	5.65	5.82	15.27	3.97	8.33	3.98	
**d.f.**	10	15	6	6	7	7	8	6	8	6	6	9	
**p**	NS	NS	NS	<0.01	NS	NS	NS	NS	NS	NS	NS	NS	

* Number of sequenced goats from each breed.

M. Moncaina; P. Pirenaica; R. Retinta; A. Alpine; S. Saanen; S.A. Scrapie Alpine; S.S. Scrapie Saanen; S.C. Scrapie Crossbreed; Pos. Positive scrapie.

d.f. Degrees of freedom (number of comparison −1).

P<0.05 has been considered statistically significant.

N.S. Not significant.

#### Native healthy herds

In addition to the Alpine and Saanen animals, the sequencing study was developed in herds belonging to three native goat breeds: Pirenaica, Moncaina and Retinta. Polymorphisms were detected at positions G37V, G74D, G127S, M137I, R139S, L141F, I142M, H143R, R151H, R154H, R211Q, Q215R, Q222K and P240S (see Table 2), and 16 out of the 18 possible haplotypes (see Table 5) were detected in the analysed populations.

Codons 142, 154, 211 and 240 have been the most variable in these populations, with frequencies ranging around 0.500 of each allele in the case of codon 240, and 0.800–0.200 for the other polymorphic codons. Rare mutations detected in codons 37, 74, 127, 137, 139, 141, 143, 151 and 215 are mostly infrequent and related with one or two subpopulations.

Only codon 142 in the Pirenaica goats and codon 211 in the Retinta goats showed frequencies higher than 0.2 for the less-common alleles (Met and Gln, respectively) (see Table 2). A new silent mutation at codon 122 (gga→ggg) was detected in the Retinta breed. This initial study indicates that a high number of polymorphic sites can be found in the goat *PRNP* gene in native populations; however, the frequencies for the rare alleles are low.

## Discussion

The first case of goat scrapie in Spain was diagnosed in 2002. In contrast with the well-studied pathological changes of ovine scrapie, the peripheral distribution of PrP^sc^ and the *PRNP* genetic background in the caprine species are not well known.

In this study, 6 out of 13 scrapie-affected animals were detected by active surveillance and 5 out of 7 by RAMALT biopsy, which demonstrates that passive surveillance is not as effective as we might expect. The reasons for this under-detection of the scrapie-affected goats could be related with the insidious characteristics of the clinical signs in this species [1] or to the presence of certain polymorphisms, such as G127S [15], which could reduce the manifestation of the clinical disease. Despite the mild clinical presentation of disease in goats, the peripheral distribution of PrP^sc^ in this species resembles that of scrapie in sheep [37], [38], [39]. The presence of PrP^sc^ in the fascicular region of the adrenal gland could suggest a haematogenous pathway of propagation for the agent because of the characteristic irrigation of this organ. Bearing in mind that the adrenal gland is an endocrine organ and that hormones must be released directly into the blood flow (estimated at approximately 7 to 17 ml per minute [40]), this gland is an over-irrigated organ. The fascicular region is full of sinusoidal capillaries between the cell layers that could contribute to increase the PrP^sc^ deposition in the cells that surround the connective tissue of the cords because of the blood-circulating prions. While the possibility of a nervous pathway, as described by other authors [37], could not be discarded, the risk of blood circulating prions should also be taken into consideration [41]. The detection of PrP^sc^ in the lymphoid tissue with absence in the ENS in one scrapie affected goat could also be explained by the relevance of these circulating prions. The presence of PrP^sc^ in the nasal mucosa has also been described in natural sheep scrapie [38]; nevertheless, in our study, PrP^sc^ was detected in the submucosa of the vestibular region of the nasal cavity where the axon bundles are located. The vestibular region is situated at the very beginning of the nasal cavity, and the presence of PrP^sc^ at this location could indicate a possible implication of the nasal route in scrapie transmission. In addition, we have seen deposition of PrP^sc^ in the lamina propria of the tongue, and although the tongue contains many innervations, PrP^sc^ seems to be aggregated throughout small clusters of lymphoid cells or sprinkled lymphocytes. As some authors have postulated, this finding could be related with the replication of PrP^sc^ when chronic inflammatory lesions are present [42]. Nevertheless, this finding also could be explained as replication of PrP^sc^ on the diffuse lymphoid aggregates present in the tongue of ruminants [43]. The relevance of the presence of PrP^sc^ in the peripheral tissues lies in its relation with specific risk material; transmission of the disease; target organs for diagnosis or different pathways of replication and transportation of prions in the animal body. In our study, the presence of PrP^sc^ in the tongue and in the nasal mucosa are relevant not only for defining risk factors in the transmission of the disease, but also as possible target tissues for applying preclinical diagnosis of scrapie.

The detection of PrP^sc^ in lymphoid biopsies as well as the distribution of PrP^sc^ in the peripheral organs suggests the absence of atypical forms of scrapie such as Nor98. Nevertheless, to ensure that BSE was not present in the TSE-affected goats further immunochemical studies were performed. The antibody battery employed in this study indicated that there were no significant differences in the intraneuronal PrP^sc^ deposition when P4 and 12B2 were compared against R-522 and 6C2. All cases were established as classical scrapie, even though the epitope sequence of the antibodies that start (P4; 93–99) or finish (6C2; 117–130) the sequence showed an apparent loss of intraneuronal staining (see Figure 2). The antibodies are focused mainly on the N-terminal globular domain (P4 93–99; R522-7 94–105; 12B2 94–105) and the hydrophobic region (1E4 108–109 and 6C2 117–130) of the prion protein [31], and subsequently, intraneuronal cleavage may be occurring at diverse sites in the sequence in different natural classical scrapie strains [44]. Despite this, none of the cases were compatible with the presence of BSE.

All these findings were confirmed by immunoblotting techniques that revealed high affinity of PrP^sc^ to both antibodies 6H4 and P4 and absence of atypical forms of scrapie. This finding is in accordance with the PrP^sc^ distribution observed all along the lymphoid tissue (biopsies and LRS) and other tissues (enteric nervous system (ENS), adrenal gland, nasal mucosa and tongue) because atypical scrapie is not related with a widespread distribution of PrP^sc^ [45] or great deposits of PrP^sc^ in the obex [2].

The *PRNP* genetic analysis of these scrapie-affected animals and its comparison with healthy animals from affected flocks or from breed survey was one of the principal objectives of this study. Here, we present five new polymorphisms of the *PRNP* gene that lead to amino acid changes (G74D, M112T, R139S, L141F and Q215R) and a new silent mutation at codon 122. Regarding the known polymorphisms that are related with scrapie susceptibility, one scrapie-affected goat of our study showed dimorphism at codon 127, which has been related with certain resistance to the development of the clinical disease [15]. Nevertheless, this animal was 8 years old when it died and showed clinical signs compatible with scrapie for only the last 6 months of life. Therefore, a delay in the development of the clinical signs is the most likely explanation for this result. Polymorphism at codon 142 has shown a certain protective effect against scrapie in goats [12]; however, 2 out of 13 scrapie-affected goats in this study displayed the I142M genotype, suggesting that methionine homozygous carriers are not absolutely protected against the disease. The mutation observed at codon 211 has been previously described in goats [46], [47], in sheep [48] and even in sheep affected with classical scrapie [33]. This mutation corresponds to the human codon 208 (R→H) (see Table 3), which is associated with Creutzfeldt-Jakob disease in homozygous 129MM patients [49]. This mutant allele showed relatively high frequency in the scrapie herds, and three scrapie-affected goats were heterozygous Q211R for this codon. This finding could indicate a certain susceptibility of the mutant allele carriers to goat TSE. As the number of scrapie animals is reduced, further analysis using experimental challenge of carrier goats or *in vitro* assays could elucidate the role of this mutation in scrapie susceptibility.

The mutated codon 222 corresponds to the human mutation Q219K. Although this mutation seems to have no consequences in human health (88% of the Japanese population carry the mutant allele [50]), the association between this polymorphism and the mutation of codon 102 has been linked to Gerstmann-Sträussler-Scheinker syndrome susceptibility. Although this polymorphism has been observed in the Moncaina, Alpine and Saanen breeds, and five heterozygous animals have been detected in the Alpine positive herd, all the scrapie-affected goats were homozygous for the wild-type allele (222QQ). This finding is in accordance with those of other authors and indicates the polymorphism Q222K is the target for the implementation of genotyping surveillance in goats [51].

The *PRNP* gene of 1259 goats has been analyzed in this study. The complexity of the genetic profile of the breeds analysed is in concordance with studies presented in other European or Asiatic breeds [1]. Polymorphisms have been shown at 16 codons (G37V, G74D, M112T, G127S, M137I, R139S, L141F, I142M, H143R, R151H, R154H, R211Q, Q215R, Q222K, G232W and P240S), of which five polymorphisms (G74D, M112T, R139S, L141F and Q215R) have, to our knowledge, not been described before in goats. These mutations are mostly located in the hydrophobic region, helix 1 and 3 and the C-terminal region of the protein, which could be related with the final stability of the protein, at least for the mutations found in helix 3 (211 and 222), which are apparently related with susceptibility/resistance to the scrapie disease.

Regarding the new polymorphisms described in this work, codon 74 showed dimorphisms at the second position (G→A transition) leading to a novel change from glycine (GGT) to aspartic acid (GAT). This polymorphism was observed at a low frequency in the Retinta breed. The characteristic situation of this dimorphism (octapeptide region), its disruption of a glycine-rich region and the change of a non-polar amino acid to a negatively charged one could contribute to the stability of the protein and subsequently to its susceptibility to change. The dimorphism present in codon 112 has been identified in sheep [52] but not in goats to date. This polymorphism has an apparently protective effect in sheep experimentally inoculated with BSE [53] or scrapie [54], but in this study, it was detected in a scrapie-affected goat with no other *PRNP* polymorphism. The relevance of this finding has to be cautiously interpreted because it has been detected just in one scrapie-affected goat; nevertheless, a complete protective effect can be discarded in this species. The polymorphic codon 139 showed a G→C transversion in the third position of the codon, inducing an amino acid change from arginine (AGG) to serine (AGC). This polymorphism, together with the glutamine (CAA) – arginine (CGA) transition shown at codon 215, showed low frequencies, and their implication in the susceptibility to scrapie disease could be evaluated if a large number of animals were analysed. The dimorphism showed in codon 141 has also been detected previously in sheep L141F [55] and, in fact, is strongly related with Nor98 susceptibility in this species [56]. We found this CTT→CTC transition with low frequency in the Moncaina breed.

Statistical analysis between the healthy Alpine and Saanen goats and the scrapie-affected animals revealed no significant differences nor did the comparison between the healthy herds and those where the scrapie outbreaks were detected. The comparison between native goat and scrapie-affected herds (data not shown) revealed differences between breeds. For example, the Pirenaica herd showed significant differences (p<0.01) compared against the Alpine, Saanen and scrapie-affected herds. This finding was also observed when this population was compared to the scrapie-affected animals. These differences were related to the high variability of codon 142 in this breed together with the fixed amino acid proline at codon 240. The Retinta breed also showed significant differences when compared with the Alpine breed. Although it has been reported that this breed is a descendant of Alpine animals [26], this finding enforces their inconsistent phylogeographical origin.

In brief, we report here the PrP^sc^ peripheral distribution in scrapie -affected goats reared in Spain, which has been characterised by the first description of PrP^sc^ in the nasal mucosa and tongue. The discriminatory study showed that neither immunohistochemical nor western blot data support the evidence for atypical scrapie or BSE in this population. Finally, we report for the first time the *PRNP* variants present in Spanish goats (scrapie-affected, healthy exposed and unexposed) revealing the complex genetics present in the native populations. Nevertheless, the presence of polymorphisms in target codons, such as 127, 142, 154, 211 and 222, do not exclude the possibility of applying a specific breeding program for the control of the scrapie disease in this country.
